# The global burden of cardiovascular diseases attributable to high body mass index, 1990–2023

**DOI:** 10.3389/fcvm.2026.1689708

**Published:** 2026-02-13

**Authors:** Manqi Sun, Yue Shi, Na Li, Ziyang Zhou, Jiahong Luo, Zaozao Chen, Duo Lin, Rubin Tan

**Affiliations:** 1 School of Public Health, Xuzhou Medical University, Xuzhou, China; 2 Department of Physiology, Basic Medical School, Xuzhou Medical University, Xuzhou, China; 3 School of Basic Medical Sciences, Southwest Medical University, Luzhou, China; 4 Department of Cardiology, Institute of Cardiovascular Research, The Affiliated Hospital, Southwest Medical University, Luzhou, China; 5 College of Life Science, Xuzhou Medical University, Xuzhou, China; 6 Department of Physiology, School of Basic Medical Sciences, Southwest Medical University, Luzhou, China

**Keywords:** body mass index, cardiovascular diseases, disability-adjusted life years, global burden of disease, regional disparities

## Abstract

**Importance:**

Obese patients are mostly accompanied by various cardiovascular diseases (CVDs), which is a major global health challenge and has important clinical significance. The global burden of CVDs caused by high body mass index (BMI) is still not well known.

**Objective:**

To determine the global burden of high BMI-related CVDs from 1990 to 2023.

**Methods:**

Following the methodological framework and analytical strategy used in the 2023 global burden of disease study (GBD 2023), we examined the death and disability-adjusted life years (DALYs) of CVDs associated with high BMI in the global population considering year, gender, age, socio-demographic index (SDI), income, and geographic location. We also calculated the annual percentage change (APC) to quantify the trends over time.

**Results:**

From 1990 to 2023, high BMI significantly contributed to over a 2-fold increase in global deaths and DALYs due to CVDs, particularly affecting ischemic heart disease (IHD), hypertensive heart disease (HHD), ischemic stroke (IS), and intracranial hemorrhage. IHD saw a doubling of deaths and DALYs, with males being more affected, and the age group with the highest deaths in over 70 years old, while DALYs peaked in the 50–69 age group. The deaths of IHD moved from high SDI to upper-middle SDI countries. Deaths and DALYs for HHD, IS, and intracranial hemorrhage also doubled, with females being slightly more affected. These conditions maintained their highest death in those over 70, but DALYs were highest in the 50–69 age group. The fastest-growing APC of deaths and DALYs were observed in low SDI countries and regions, as well as lower-middle income countries and regions, indicating a significant shift in the geographic distribution of CVD death.

**Conclusion:**

High BMI is a major contributor to CVDs globally, leading to significant increases in deaths and DALYs over the past three decades. The impact varies by age, gender, income level, and geographic region, with the APC growing most rapidly in lower-middle income populations. IHD is particularly concerning, with notable gender and age-specific trends. Addressing high BMI and its associated cardiovascular risks, especially in vulnerable populations, is crucial to mitigating the growing impact of obesity-related CVDs.

## 1 Introduction

Cardiovascular diseases (CVDs), have become a major public health challenge, with WHO data indicating in 2022, nearly 20 million deaths were attributed to CVDs, representing approximately 32% of all global deaths (1). The increasing prevalence of high body mass index (BMI) is a significant contributor to this trend, with 2.2 billion adults worldwide classified as having a high BMI in 2020, a number expected to rise to 3.3 billion by 2035, or 54% of the global adult population (2). Among children aged 5–19, the proportion with a high BMI is projected to increase from 22% to 39% by 2035 (2). High income regions like North America and parts of Europe have particularly high rates of high BMI among adults (3). The issue affects all age groups, with a notable increase in adolescents, and is economically pronounced in lower-middle income countries. High BMI individuals are at a higher risk of developing various diseases, including CVDs, type 2 diabetes, and chronic kidney disease, underscoring the urgency for in-depth research into CVDs and their risk factors (2, 4).

Unhealthy lifestyle habits like high-calorie diets, lack of exercise, smoking, and sedentary behavior contribute to the rise in high BMI and are key risk factors for CVDs (5–7), including ischemic heart disease (IHD), ischemic stroke (IS), and coronary artery disease, are leading causes of global death, challenging the health system (8, 9). Understanding the link between high BMI and CVDs is crucial for developing prevention strategies. The global burden of disease(GBD), injuries, and risk factors study 2023 data, released in 2025, provides a comprehensive assessment of the global impact of health challenges, including the burden of CVDs attributable to high BMI, such as IHD, hypertensive heart disease (HHD), IS, and intracerebral hemorrhage, over the past decades (10). This information is vital for crafting effective public health interventions to address the escalating threat of CVDs associated with high BMI.

This study aims to utilize the latest GBD data to analyze, for the first time, the relationship between the high BMI population and several common CVDs, as well as the global burden, during the period from 1990 to 2023. Special emphasis will be placed on regional, age, and gender differences. Through this research, we expect to provide a scientific basis for the prevention and control of CVDs among the global high BMI population and offer support for formulating effective public health strategies.

## 2 Methods

### 2.1 Data sources

The GBD 2023 study is a comprehensive multinational research estimating global burden using data from various sources and tools (http://ghdx.healthdata.org/gbd-results-tool). It analyzed burden globally, regionally, and nationally, stratified by socio-demographic index (SDI). Disability-adjusted life-years (DALYs) measure healthy life years lost due to disease, including years of life lost (YLL) and years lived with disability (YLD). The study provided estimates for incidence, prevalence, DALYs, and death for each disease and risk factor, focusing on CVDs related to high BMI.

### 2.2 Definitions in GBD 2023

The four-level classification system for risk factors in the GBD 2023 is as follows: the first level is divided into three major categories, namely behavioral, metabolic, and environmental and occupational factors; the second level covers 20 risk clusters; the third level consists of 42 specific risk factors; and the fourth level includes 22 distinct factors. Notably, the metabolic category does not involve further subdivision into the third and fourth levels. Metabolic risk factors encompass high fasting plasma glucose, high systolic blood pressure, high BMI, low bone mineral density, kidney dysfunction, and high low density lipoprotein cholesterol. In total, this classification system encompasses 88 modifiable risk factors. Further details on these five risk factors are available in the GBD 2023 online resources (https://ghdx.healthdata.org/gbd-2023/sources?components.6&risks.367&location). It examines the global burden of CVDs attributable to high BMI across all ages, using deaths and DALYs as metrics. The cause-of-death ensemble model estimates high BMI-attributable deaths by incorporating predictive covariates, accounting for location, age, sex, and year.

### 2.3 Deaths, DALYs, and annual percentage change (APC)

From 1990 to 2023, the GBD study measured the global, regional, and national burden of metabolic diseases using deaths and DALYs for all age groups. It assessed the impact of high BMI on global burden by calculating potential DALY changes at the theoretical minimum risk level. The GBD 2023 Disease and Injury Collaborators performed 500 iterations for each calculation to produce sample-level estimates, reporting the meaning of these samples as the final estimate. The 95% uncertainty intervals were determined by the 2.5th and 97.5th percentiles of the samples. Data visualization and tabular results (https://vizhub.healthdata.org/gbd-results/) are available online, with analyses following GBD's established rules and procedures. The APC is a widely used indicator to measure the trend of standardized rates over a specific period, and was calculated by Joinpoint regression analysis.

## 3 Results

### 3.1 Overview

**Figure 1:**
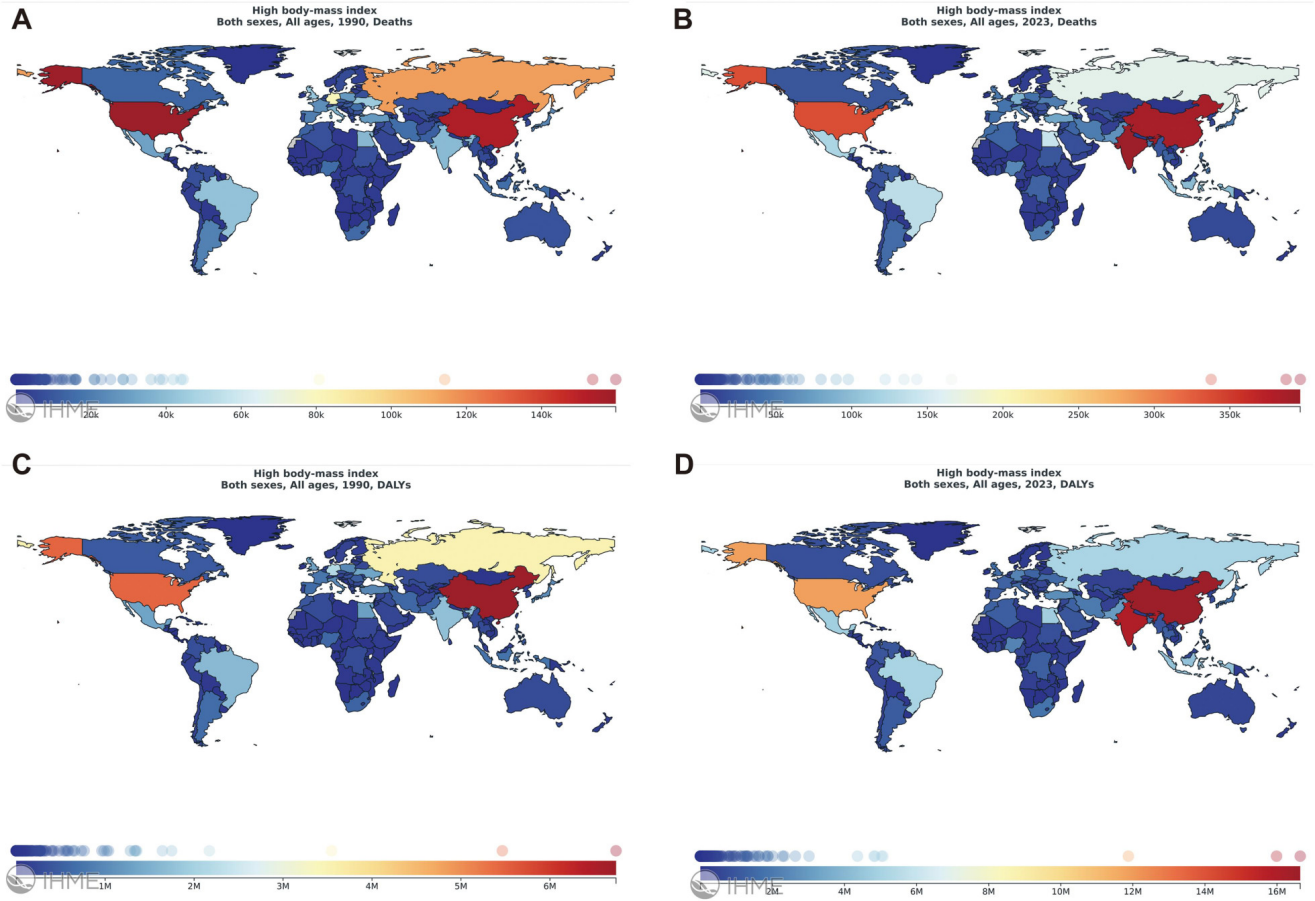
Global distribution of disease burden of high BMI in 1990 and 2023. Global maps of high BMI deaths **(A)** and DALYs **(C)** in 1990, and deaths **(B)** and DALYs **(D)** in 2023. Maps reproduced from the GBD database, Institute for Health Metrics and Evaluation (https://vizhub.healthdata.org/), licensed under CC BY-NC-ND 4.0.

In 2023, the numbers of deaths and DALYs of all disease sub-categories increased by 1–3 times compared to 1990 (Supplementary Table S1). Among diseases attributable to high BMI, CVDs account for the highest of deaths, diabetes and kidney diseases account for the highest of DALYs. In 1990, Central Europe, Eastern Europe and Central Asia were the regions with the top of deaths and DALYs attributable to high BMI. By 2023, the region with the top of deaths remained unchanged, while China became the territory with the highest of DALYs. (Figure 1). In 2023, the other two territories with the top of deaths also included India and China. Further ranking analysis of deaths and DALYs of various CVDs caused by BMI showed that the top four were IHD, HHD, IS, and intracerebral hemorrhage. The rankings remained stable in 1990 and 2023 (Supplementary Figure S1). Therefore, we mainly analyzed the impact of BMI on the global burden of these four CVDs.

### 3.2 Ischemic heart disease

IHD has the heaviest burden among high BMI-related CVDs. It has a high probability of causing heart failure (11). Among middle-aged and older adult people, the numbers of deaths caused by it shows a trend of getting younger. Tobacco, hypertension, hyperlipidemia, and high BMI are the main risk factors for IHD (12, 13). In 2023, 8.90 million (95% UI: 8.04–9.65) deaths were related to IHD, and 0.94 million (95% UI: 0.43–1.47) deaths were related to IHD caused by high BMI, accounting for 10.6%. In 2023, it was estimated that 24.56 million (95% UI: 11.44–37.26) DALYs of all age groups were caused by IHD due to high BMI, doubling compared to 33 years ago (Table 1). Males accounted for 59.6% (14.65 million) of these DALYs, and females accounted for 40.4% (9.91 million). Among all age groups, the over 70 years old group had the highest of deaths [0.45 million (95% UI: 0.20–0.73)], and the 50–69-year-old group had the highest of DALYs [12.25 million (95% UI: 5.81–18.26)].

**Table 1:** The burden attributable to BMI risks in ischemic heart disease in 1990 and 2023 and the temporal trends from 1990 to 2023.

Demographics	1990 deaths (95% UI)	2023 deaths (95% UI)	Annual percentage c hange of death rates (95% CI)	1990 DALYs (95% UI)	2023 DALYs (95% UI)	Annual percentage change of DALYs^a^ rates (95% CI)
Global	491,930 (219,883-813,116)	938,363 (426,018–1,470,248)	1.7647 (1.6945–1.8350)	12,682,691 (5,679,998–20,662,581)	24,557,447 (11,439,189–37,262,291)	1.7985 (1.7263–1.8708)
Sex						
Male	254,992 (114,705–414,176)	516,375 (242,351–793,280)	1.9656 (1.8913–2.0400)	7,317,284 (3,356,912–11,715,075)	14,646,197 (6,978,115–22,045,850)	1.9177 (1.8484–1.9871)
Female	236,938 (102,751–396,456)	421,988 (183,667–667,818)	1.5381 (1.4636–1.6126)	5,365,407 (2,338,330–8,765,573)	9,911,250 (4,439,885–15,206,989)	1.6313 (1.5515–1.7112)
Age groups						
0–14						
15–19						
20–24	1,025 (418–1,853)	2,129 (920–3,570)	2.1816 (2.0671–2.2962)	73,666 (29,725–132,270)	153,220 (65,707–258,032)	2.1956 (2.0810–2.3102)
25–29	2,085 (952–3,478)	4,436 (1,916–7,121)	2.2001 (2.0378–2.3627)	142,046 (63,753–237,540)	300,214 (128,644–480,467)	2.1991 (2.0400–2.3583)
30–34	4,642 (2,104–7,530)	10,267 (4,615–16,038)	2.2222 (2.1254–2.3191)	288,586 (128,618–469,496)	634,748 (283,399–987,147)	2.5421 (2.4655–2.6188)
35–39	8,576 (3,940–13,543)	17,854 (8,030–27,040)	1.8124 (1.6896–1.9353)	486,025 (219,939–781,550)	1,009,819 (448,669–1,519,720)	1.8155 (1.6981–1.9330)
40–44	13,620 (6,410–21,503)	29,734 (14,541–44,199)	1.8080 (1.6321–1.9843)	694,304 (322,925–1,103,798)	1,507,769 (727,476–2,250,245)	1.8030 (1.6294–1.9770)
45–49	18,881 (8,937–29,935)	42,784 (20,553–62,470)	2.1594 (1.8821–2.4375)	862,618 (405,570–1,374,058)	1,951,727 (938,289–2,861,312)	2.1473 (1.8709–2.4243)
50–69	204,176 (92,407–326,843)	383,539 (181,459–569,085)	1.7602 (1.6071–1.9135)	6,449,144 (2,896,711–10,275,035)	12,249,546 (5,809,221–18,256,910)	1.8473 (1.7224–1.9723)
Over 70	238,925 (102,796–403,195)	447,620 (197,284–732,738)	1.7138 (1.6365–1.7912)	3,686,301 (1,580,516–6,163,962)	6,750,405 (2,978,530–10,750,104)	1.5510 (1.4402–1.6619)
SDI Quintile						
Low SDI	10,806 (4,104–20,053)	62,132 (25,969–105,305)	5.7874 (5.6137–5.9615)	347,297 (132,933–639,688)	2,009,013 (844,924–3,342,068)	5.8000 (5.6325–5.9678)
Low-middle SDI	22,444 (9,551–37,598)	95,494 (43,188–153,982)	4.6896 (4.5715–4.8079)	690,488 (295,941–1,150,207)	2,853,098 (1,290,312–4,547,137)	4.6298 (4.4994–4.7604)
Middle SDI	31,868 (13,675–52,104)	139,213 (66,398–212,979)	4.5278 (4.4412–4.6144)	977,595 (423,151–1,586,487)	4,120,262 (1,979,735–6,235,200)	4.4598 (4.3565–4.5633)
High-middle SDI	46,019 (20,485–75,968)	168,313 (76,527–265,193)	4.1739 (4.0715–4.2763)	1,418,525 (628,482–2,363,057)	4,642,724 (2,111,832–7,044,349)	3.7772 (3.6810–3.8734)
High SDI	379,837 (169,106–630,100)	471,989 (222,434–737,602)	0.3091 (0.1887–0.4298)	9,224,317 (4,187,010–15,032,997)	10,902,436 (5,231,599–16,289,577)	0.0885(−0.0537–0.2309)
World Bank income level						
High income	295,240 (133,011–482,529)	299,466 (141,739–471,246)	−0.3197(−0.44499 to −0.1893)	6,929,834 (3,165,445–10,961,754)	6,660,372 (3,212,611–10,044,400)	−0.5429(−0.6942 to −0.3914)
Upper-middle income	136,952 (62,099–226,713)	350,776 (160,784–555,436)	2.6713 (2.5843–2.7584)	3,889,163 (1,781,712–6,355,528)	9,185,540 (4,321,525–13,863,294)	2.3835 (2.2901–2.4770)
Lower-middle income	47,830 (20,027–81,049)	252,794 (116,577–398,778)	5.4370 (5.2386–5.6357)	1,511,958 (641,987–2,561,157)	7,676,138 (3,539,986–12,013,066)	5.3101 (5.0904–5.5303)
Low income	10,948 (4,577–18,494)	34,098 (14,864–57,523)	3.2739 (3.1330–3.4149)	327,135 (138,015–539,134)	1,005,269 (441,406–1,676,051)	3.2480 (3.1127–3.3836)
Countries and territories^b^						
Central Europe, Eastern Europe and Central Asia	157,863 (69,505–264,277)	192,506 (88,690–299,842)	0.0808(−0.2199–0.3823)	3,891,555 (1,744,131–6,386,996)	4,348,613 (2,032,096–6,548,156)	−0.3525(−0.6808 to −0.0232)
China	31,193 (13,467–53,024)	114,980 (51,394–191,636)	3.9748 (3.8089–4.1409)	1,033,065 (453,634–1,735,646)	2,923,595 (1,313,596–4,744,941)	2.9858 (2.8457–3.1261)
India	9,978 (3,765–18,352)	88,634 (39,361–144,517)	7.7901 (7.1410–8.4431)	349,438 (133,114–634,768)	2,769,518 (1,213,140–4,451,795)	7.2924 (6.6598–7.9288)
USA	71,388 (32,867–112,391)	80,286 (37,224–125,780)	0.1729 (0.0527–0.2932)	1,673,480 (786,346–2,593,264)	1,874,938 (885,193–2,812,076)	0.2294 (0.1346–0.3244)
Russian Federation	61,619 (27,821–99,316)	76,253 (35,049–119,295)	−0.0665(−0.5500–0.4194)	1,512,116 (703,620–2,407,456)	1,733,036 (804,696–2,658,161)	−0.5461(−1.0755 to −0.0138)
Mexico	4,691 (2,122–7,429)	21,468 (10,151–33,137)	4.6771 (4.4116–4.9432)	135,363 (61,535–207,262)	573,567 (277,532–849,823)	4.5014 (4.2614–4.7419)
Indonesia	2,447 (963–4,493)	20,191 (8,711–33,348)	7.3113 (7.0655–7.5576)	90,322 (35,950–164,185)	753,813 (330,533–1,230,623)	7.4245 (7.1360–7.7139)

APC, annual percentage change (APC is used to represent the trend of rate. If APC > 0 and lower UI > 0, upward trend; if APC < 0 and upper UI < 0, downward trend).

DALYs, disability-adjusted life years; SDI, socio-demographic index; UI, uncertainty interval.

a Age-standardized DALYs (or death) per 100,000 population.

b The top 7 locations with the highest numbers of DALYs or deaths.

The death and the DALYs were the highest in high SDI [0.47 million (95% UI: 0.22–0.74) for deaths, 10.90 million (95% UI: 5.23–12.69) for DALYs]. The number of deaths was the highest among the upper-middle income population [0.35 million (95% UI: 0.16–0.56)], as well as the number of DALYs [9.19 million (95% UI: 4.32–13.86)]. In 2023, the number of IHD deaths in China [0.11 million (95% UI: 0.05–0.19)] exceeded that of the United States of America (USA) [0.08 million (95% UI: 0.04–0.13)]. Meanwhile, Central Europe, Eastern Europe and Central Asia had the top of DALYs of IHD over 33 years ago [4.35 million (95% UI: 2.03–6.55)] (Supplementary Figure S2). The other three territories with the top numbers of DALYs of high BMI-related IHD in 2023 were China [2.92 million (95% UI: 1.31–4.74)], India [2.77 million (95% UI: 1.21–4.45)], and the USA [1.87 million (95% UI: 0.89–2.81)].

### 3.3 Hypertensive heart disease

Research shows that an increase in BMI leads to fat accumulation in the body, triggering a series of metabolic disorders and activating the renin-angiotensin-aldosterone system, which promotes an increase in blood pressure (14). The long-term state of high blood pressure increases the afterload of the heart, causing myocardial cells to undergo compensatory hypertrophy and gradually develop into HHD (15). The probability of high BMI people suffering from hypertension is several times higher than that of people with normal weight, which significantly increases the risk of HHD (16).

In 2023,1.48 million (95% UI: 1.18–1.82) deaths were related to HHD, and 0.60 million (95% UI: 0.37–0.81) deaths were related to HHD caused by high BMI, accounting for 40.5%. In 2023, it was estimated that 13.18 million (95% UI: 10.09–16.94) DALYs of all age groups were caused by HHD due to high BMI. Males accounted for 46.6% (6.14 million) of these DALYs, and females accounted for 53.4% (7.04 million) (Table 2). Among all age groups, the number of deaths was the highest among those over 70 years old [0.37 million (95% UI: 0.17–0.56)], and the number of DALYs was the highest in the 50–69-year-old group [5.70 million (95% UI: 4.09–7.63)].

**Table 2:** The burden attributable to BMI risks in Hypertensive heart disease in 1990 and 2023 and the temporal trends from 1990 to 2023.

Demographics	1990 deaths (95% UI)	2023 deaths (95% UI)	Annual percentage change of death rates (95% CI)	1990 DALYs (95% UI)	2023 DALYs (95% UI)	Annual percentage change of DALYs^a^ rates (95% CI)
Global	214,447 (146,077–296,896)	601,860 (373,972–811,176)	3.2732 (3.1220–3.4246)	5,170,755 (3,761,998–6,750,563)	13,182,187 (10,092,464–16,940,989)	2.9601 (2.8161–3.1043)
Sex						
Male	99,844 (66,147–138,048)	250,566 (166,735–330,627)	3.0200 (2.8548–3.1856)	2,652,707 (1,870,609–3,645,694)	6,138,169 (4,516,940–7,974,568)	2.7466 (2.6117–2.8817)
Female	114,602 (70,453–166,754)	351,294 (203,071–506,781)	3.5067 (3.3686–3.6450)	2,518,047 (1,674,938–3,465,003)	7,044,018 (4,899,741–10,080,866)	3.1575 (3,0047–3.3104)
Age groups						
0–14						
15–19						
20–24	560 (250–1,010)	859 (433–1,396)	1.3310 (1.1927–1.4695)	39,448 (17,603–69,971)	61,294 (31,080–97,182)	1.3894 (1.2550–1.5240)
25–29	979 (453–1,592)	1,782 (909–2,829)	1.7188 (1.5493–1.8885)	64,196 (29,844–103,444)	117,109 (62,117–182,032)	1,7645 (1.6027–1.9265)
30–34	1,911 (976–2,981)	4,441 (2,582–6,912)	2.4439 (2.3143–2.5736)	113,828 (58,512–176,408)	264,582 (153,209–408,769)	2.4630 (2.3417–2.5843)
35–39	3,015 (1,721–4,483)	7,381 (4,504–10,604)	2.6167 (2.4835–2.7501)	163,502 (93,922–241,933)	401,133 (248,840–573,471)	2.6316 (2.5042–2.7592)
40–44	4,822 (2,868–6,848)	12,398 (8,155–18,065)	2.6219 (2.5420–2.7020)	236,088 (140,690–334,508)	608,555 (406,452–884,181)	2.6365 (2.5581–2.7150)
45–49	7,188 (4,034–10,437)	19,229 (12,776–26,645)	3,0116 (2.9029–3.1204)	315,845 (179,038–459,228)	849,834 (569,997–1,175,381)	3.0267 (2.9171–3.1364)
50–69	83,529 (55,092–114,008)	185,611 (133,523–252,246)	2.5856 (2.3324–2.8395)	2,510,820 (1,667,404–3,360,593)	5,696,879 (4,093,686–7,634,125)	2.6901 (2.4581–2.9225)
Over 70	112,444 (60,854–174,697)	370,159 (172,918–556,463)	3.8172 (3.7077–3.9268)	1,727,028 (1,058,735–2,647,944)	5,182,800 (2,896,270–7,409,397)	3.4422 (3.3484–3.5360)
SDI Quintile						
Low SDI	15,889 (8,968–25,480)	82,467 (47,937–129,697)	5.1794 (5.0376–5.3213)	454,078 (267,808–733,213)	2,257,382 (1,401,774–3,484,547)	5.0589 (4.8978–5.2204)
Low-middle SDI	15,806 (9,614–24,422)	66,830 (43,406–101,351)	4.6238 (4.5247–4.7230)	415,437 (263,798–615,420)	1,669,228 (1,133,107–2,456,128)	4.4837 (4.3913–4.5763)
Middle SDI	25,031 (16,388–34,983)	88,791 (62,417–119,486)	3.8765 (3.8252–3.9279)	664,772 (446,335–893,285)	2,209,590 (1,587,045–2,943,718)	3.7052 (3.6640–3.7464)
High-middle SDI	51,440 (32,084–75,234)	120,641 (73,858–169,037)	2.7138 (2.2857–3.1437)	1,303,428 (820,550–1,841,497)	2,584,273 (1,890,731–3,341,494)	2.3376 (1.9273–2.7496)
High SDI	105,907 (67,113–142,748)	242,183 (128,645–343,863)	2.7408 (2.6397–2.8419)	2,324,748 (1,765,570–2,887,816)	4,442,606 (3,134,349–5,588,949)	2.0914 (2.0189–2.1640)
World Bank income level						
High income	63,525 (38,047–89,281)	162,991 (82,886–227,796)	3.3258 (3.1611–3.4907)	1,289,744 (940,102–1,619,687)	2,909,160 (2,033,267–3,632,001)	2.8375 (2.6695–3.0059)
Upper-middle income	102,991 (67,542–147,508)	210,406 (123,501–288,955)	2.1103 (1.7654–2.4563)	2,574,710 (1,744,292–3,525,297)	4,432,681 (3,213,309–5,615,959)	1.6960 (1.3685–2.0246)
Lower-middle income	36,499 (21,861–54,804)	184,781 (119,370–271,238)	5.2113 (5.1317–5.2910)	995,001 (613,743–1,444,292)	4,686,598 (3,167,945–6,525,829)	4.9918 (4.9141–5.0695)
Low income	11,051 (6,252–18,592)	42,724 (24,570–69,039)	3.9692 (3.7796–4.1591)	302,797 (169,793–509,326)	1,134,383 (656,920–1,763,725)	3.9103 (3.6872–4.1338)
Countries and territories^b^						
China	59,391 (32,515–97,034)	89,723 (44,581–131,963)	0.9311 (0.1443–1.7240)	1,514,549 (816,811–2,359,768)	1,766,267 (1,114,172–2,393,054)	0.3808 (−0.3348–1.1015)
Central Europe, Eastern Europe and Central Asia	25,822 (17,963–33,473)	54,082 (33,211–72,060)	2.7105 (2.2676–3.1553)	583,600 (456,080–708,834)	1,006,385 (752,324–1,225,621)	1.8607 (1.3855–2.3382)
India	6,639 (3,072–11,904)	64,203 (35,958–106,835)	7,8590 (7.4922–8.2271)	190,195 (88,716–327,308)	1,584,467 (933,109–2,585,683)	7.2941 (6.9596–7.6295)
USA	12,071 (8,120–15,396)	50,948 (33,504–65,021)	4.6121 (4.4818–4.7424)	291,212 (235,206–335,696)	1,133,693 (905,571–1,314,562)	7.2941 (6.9596–7.6295)
Indonesia	3,532 (1,656–6,299)	17,737 (9,222–27,762)	5.2701 (5.1434–5.3970)	111,841 (53,867–191,273)	560,973 (299,396–884,914)	5.3872 (5.2352–5.5394)
Mexico	2,028 (1,251–2,797)	5,796 (3,458–7,683)	3.4431 (3.1285–3.7588)	42,265 (32,008–52,355)	111,768 (81,368–135,431)	3.2716 (2.9018–3.6428)

APC, annual percentage change (APC is used to represent the trend of rate. If APC > 0 and lower UI > 0, upward trend; if APC < 0 and upper UI < 0, downward trend).

DALYs, disability-adjusted life years; SDI, socio-demographic index; UI, uncertainty interval.

a Age-standardized DALYs (or death) per 100,000 population.

b The top 6 locations with the highest numbers of DALYs or deaths.

From 1990 to 2023, the DALYs of HHD caused by high BMI globally increased 2.5 times that of 33 years ago. Over the past 33 years, China has been the territory with the highest DALYs of HHD (Supplementary Figure S3). The top three countries and territories with the top of deaths of high BMI-related HHD were China [0.09 million (95% UI: 0.04–0.13)], Central Europe, Eastern Europe and Central Asia [0.05 million (95% UI: 0.03–0.07)], and India [0.06 million (95% UI: 0.04–0.11)]. The death and DALYs were the highest in high SDI countries and regions (0.24 million (95% UI: 0.13–0.34) for death, [4.44 million (95% UI: 3.12–5.59) for DALYs]. The number of DALYs was the highest among the lower-middle income population [4.69 million (95% UI: 3.17–6.53)], and the number of deaths was the highest among the upper-middle income population [0.21 million (95% UI: 0.12–0.29)].

### 3.4 Ischemic stroke

In 2023, 3.27 million (95% UI: 2.87–3.68) deaths were related to IS, while 0.15 million (95% UI: 0.03–0.29) deaths were caused by IS due to high BMI, accounting for 4.6%. From 1990 to 2023, the global DALYs of IS caused by high BMI increased approximately 1.75-fold (Table 3). Males accounted for 48.8% (2.00 million) of the DALYs, while females accounted for 51.2% (2.09 million) of the DALYs. Among all age groups, the number of deaths was the highest in the population over 70 years old [0.09 million (95% UI: 0.01–0.18)], and the number of DALYs was the highest in the 50–69-year-old population [1.95 million (95% UI: 0.38–3.59)].

**Table 3:** The burden attributable to BMI risks in ischemic stroke in 1990 and 2023 and the temporal trends from 1990 to 2023.

Demographics	1990 deaths (95% UI)	2023 deaths (95% UI)	Annual percentag change of death rates (95% CI)	1990 DALYs (95% UI)	2023 DALYs (95% UI)	Annual percentage change of DALYs^a^ rates (95% CI)
Global	93,441 (15,904–178,189)	146,525 (25,945–290,127)	1.0162 (0.8771–1.1556)	2,334,035 (419,564–4,272,813)	4,090,001 (768,125–7,596,196)	1.3804 (1.2561–1.5048)
Sex						
Male	37,095 (6,475–69,808)	66,854 (12,363–124,971)	1.5057 (1.3831–1.6283)	1,016,394 (181,004–1,927,634)	1,997,862 (383,460–3,632,195)	1.8041 (1.6960–1.9123)
Female	56,346 (9,850–107,040)	79,671 (13,496–156,414)	0.6605 (0.5071–0.8142)	1,317,641 (242,169–2,384,546)	2,092,139 (389,303–3,920,726)	1.0178 (0.8781–1.1577)
Age groups						
0–14						
15–19						
20–24	111 (20–240)	204 (37–416)	1.8638 (1.7076–2.0230)	12,995 (2,383–25,829)	26,971 (5,039–52,908)	2.2263 (2.0727–2.3832)
25–29	195 (39–369)	358 (71–685)	1.8691 (1.7151–2.0223)	22,380 (4,319–42,360)	45,398 (9,072–83,826)	2.1919 (2.0625–2.3214)
30–34	384 (82–720)	758 (153–1,428)	1.9572 (1.8574–2.0567)	37,352 (7,592–69,569)	80,923 (16,534–148,250)	2.2407 (2.1460–2.3340)
35–39	689 (153–1,269)	1,337 (289–2,513)	1.7405 (1.6255–1.8576)	57,044 (11,657–105,163)	125,070 (26,064–226,217)	2.1258 (2.0116–2.2403)
40–44	1,068 (223–1,924)	2,199 (438–4,060)	1.7422 (1.5421–1.9452)	72,940 (14,511–130,105)	168,956 (35,716–306,673)	2.1706 (1.9912–2.3530)
45–49	1,745 (331–3,117)	3,662 (707–6,475)	2.0038 (1.7094–2.3003)	97,941 (18,420–176,936)	232,743 (48,110–410,600)	2.4223 (2.1637–2.6877)
50–69	33,948 (6,478–61,670)	51,520 (9,838–95,099)	0.8341 (0.5652–1.1011)	1,139,690 (221,381–2,116,638)	1,951,479 (379,104–3,589,995)	1.3321 (1.1167–1.5490)
Over 70	55,301 (8,817–109,696)	86,487 (14,448–181,328)	1.0487 (0.8848–1.2108)	893,694 (144,908–1,724,451)	1,458,462 (247,996–2,986,327)	1.0833 (0.8644–1.2966)
SDI Quintile						
Low SDI	2,317 (445–5,180)	10,679 (1,636–22,272)	5.0043 (4.8596–5.1492)	70,647 (13,566–156,443)	341,341 (56,663–673,984)	5.1591 (5.0213–5.2966)
Low-middle SDI	3,888 (717–8,044)	14,163 (2,369–28,715)	4.1295 (4.0422–4.22166)	112,133 (20,959–225,401)	422,213 (77,148–811,990)	4.2778 (4.1922–4.3630)
Middle SDI	5,812 (1,068–10,981)	19,464 (3,434–37,727)	3.6595 (3.4991–3.8217)	173,986 (32,586–323,244)	600,528 (116,095–1,093,974)	3.7990 (3.6314–3.9693)
High-middle SDI	9,081 (1,578–16,875)	24,475 (4,341–50,564)	3.1552 (3.0429–3.2598)	271,015 (48,020–498,309)	736,527 (135,473–1,448,683)	3.1433 (3.0490–3.2325)
High SDI	72,150 (12,520–135,838)	77,529 (14,485–152,198)	−0.2958(−0.5190 to −0.0751)	1,701,834 (305,554–3,141,069)	1,984,240 (390,851–3,824,712)	−0.0706(−0.2951–0.1497)
World Bank income level						
High income	52,276 (9,159–98,882)	48,382 (8,849–94,898)	−0.8080(−1.0793 to −0.5388)	1,172,865 (214,680–2,171,141)	1,174,393 (228,438–2,268,207)	−0.5849(−0.8517 to −0.3259)
Upper-middle income	30,680 (5,247–58,163)	56,940 (10,714–109,664)	1.6742 (1.5767–1.7696)	850,696 (150,219–1,592,358)	1,656,460 (321,627–3,142,976)	1.7914 (1.6937–1.8852)
Lower-middle income	7,793 (1,455–15,740)	33,588 (5,706–69,350)	4.7288 (4.5829–4.8759)	234,741 (44,373–447,806)	1,032,642 (192,142–1,965,156)	4.8033 (4.6645–4.9410)
Low income	2,500 (488–5,229)	7,400 (1,280–15,891)	3.1256 (3.0122–3.2373)	71,297 (14,108–149,788)	221,336 (37,258–464,402)	3.3230 (3.1916–3.4527)
Countries and territories^b^						
China	10,233 (1,640–20,591)	23,498 (4,449–48,349)	2.2906 (2.0762–2.5007)	320,162 (52,342–647,144)	735,647 (140,937–1,458,617)	2.2745 (2.1035–2.4451)
Central Europe, Eastern Europe and Central Asia	37,674 (6,671–71,174)	34,084 (6,137–63,940)	−0.9785 (−1.3694 to −0.5878)	883,728 (159,312–1,646,867)	786,199 (149,291–1,451,521)	−1.1063 (−1.4859 to −0.7310)
USA	4,794 (860–9,430)	8,305 (1,650–16,641)	1.2644 (0.8970–1.6378)	131,629 (25,890–249,097)	266,910 (55,078–508,511)	1.9521 (1.7474–2.1606)
Russian Federation	17,993 (3,412–34,739)	16,682 (2,928–32,381)	−1.2402 (−1.8291 to −0.6567)	416,672 (80,180–790,852)	372,508 (68,804–697,977)	−1.4396 (−2.0036 to −0.8682)
India	656 (115–1,576)	8,460 (1,347–18,991)	9.2986 (8.7128–9.9333)	22,058 (3,775–51,102)	270,084 (47,280–552,436)	8.9561 (8.3989–9.5368)
Indonesia	402 (82–869)	2,970 (507–6,394)	6.7692 (6.4528–7.0833)	16,789 (3,199–32,979)	123,211 (22,743–252,430)	6.7753 (6.4732–7.0754)
Mexico	703 (120–1,327)	1,394 (285–2,622)	2.2037 (2.0377–2.3678)	20,434 (3,815–37,346)	40,706 (8,533–73,417)	2.1588 (1.9296–2.3901)

APC, annual percentage change (APC is used to represent the trend of rate. If APC > 0 and lower UI > 0, upward trend; if APC < 0 and upper UI < 0, downward trend).

DALYs, disability-adjusted life years; SDI, socio-demographic index; UI, uncertainty interval.

a Age-standardized DALYs (or death per 100,000 population.

b The top 7 locations with the highest numbers of DALYs or deaths.

The death [0.08 million (95% UI: 0.01–0.15)] and DALYs [1.98 million (95% UI: 0.39–3.82)] were the highest in high SDI. The upper-middle income population had the top numbers of deaths [0.06 million (95% UI: 0.01–0.11)] and DALYs [1.66 million (95% UI: 0.32–3.14)]. Over the past three decades, China has been the territory with the heaviest burden of IS Supplementary Figure S4). Globally, the top three countries and territories with the top numbers of deaths and DALYs of high BMI-related IS were Central Europe, Eastern Europe and Central Asia, China, and Russia.

### 3.5 Intracerebral hemorrhage

Intracerebral hemorrhage refers to the bleeding caused by the rupture of blood vessels within the non-traumatic brain parenchyma. It is an acute cerebrovascular disease characterized by high incidence, high disability rate, and high death. High BMI has a significant impact on the global burden of intracerebral hemorrhage.

In 2023, 3.16 million (95% UI: 2.75–3.55) deaths were related to intracerebral hemorrhage, while 0.07 million (95% UI: −6,116–162,868) deaths were caused by intracerebral hemorrhage due to high BMI. In 2023, it was estimated that 2.33 million (95% UI: −0.12–5.46) DALYs across all age groups were caused by intracerebral hemorrhage resulting from high BMI (Table 4). From 1990 to 2023, the global DALYs of intracerebral hemorrhage caused by high BMI increased approximately 3.13-fold. Males accounted for 45.2% (1.05 million) of the DALYs, and females accounted for 54.8% (1.28 million) of the DALYs. Among all age groups, globally, the 50–69-year-old population had the top numbers of deaths [0.04 million (95% UI: −1,383–91,102)] and DALYs [1.24 million (95% UI: −0.04–2.86)]. The death [0.03 million (95% UI: 487–58,815)] and DALYs [0.81 million (95% UI: 10,620–1,729,374)] were the highest in high SDI. The upper-middle income population had the top numbers of deaths [0.03 million (95% UI: −4,265–73,914)] and DALYs [1.06 million (95% UI: −64,348–2,415,566)].

**Table 4:** The burden attributable to BMI risks in intracerebral hemorrhage in 1990 and 2023 and the temporal trends from 1990 to 2023.

Demographics	1990 deaths (95% UI)	2023 deaths (95% UI)	Annual percentage change of death rates (95% CI)	1990 DALYs (95% UI)	2023 DALYs (95% UI)	Annual percentage change of DALYs^a^ rates (95% CI)
Global	23,286 (−17,523–64,379)	68,691 (−6,116–162,868)	2.8661 (2.7101–3.0224)	744,326 (−610,310–2,104,923)	2,331,781 (−118,188–5,460,020)	3.0809 (2.9425–3.2196)
Sex						
Male	7,159 (−15,696–27,848)	30,137 (−8,381–76,668)	3.7569 (3.5762–3.9379)	240,479 (−525,387–965,159)	1,053,214 (−260,925–2,526,417)	3.8835 (3.7058–4.0615)
Female	16,127 (−4,521–40,985)	38,554 (−434–86,562)	2.2465 (2.0950–2.3982)	503,847 (−135,646–1,272,862)	1,278,567 (15,086–2,708,471)	2.4862 (2.3565–2.6160)
Age groups						
0–14						
15–19						
20–24	−118 (−711–210)	204 (−211–618)	5.4314 (5.0641–5.8000)	−8,599 (−52,893–16,661)	16,222 (−16,180–47,181)	5.4181 (5.0485–5.7889)
25–29	73 (−561–568)	597 (−115–1,421)	5.2517 (4.9079–5.5966)	5,446 (−39,805–39,970)	43,260 (−8,457–100,044)	5.1933 (4.8574–5.5303)
30–34	354 (−659–1,310)	1,476 (−111–3,430)	4.2520 (4.1432–4.3609)	23,056 (−39,614–83,595)	94,949 (−6,762–223,494)	4.2069 (4.1000–4.3138)
35–39	817 (−864–2,715)	2,952 (−68–6,816)	3.6164 (3.5067–3.7262)	46,800 (−49,393–154,303)	169,347 (−3,706–388,270)	3.6188 (3.5141–3.7235)
40–44	1,263 (−1,273–3,713)	4,851 (−84–10,200)	3.4186 (3.2293–3.6083)	63,933 (−62,610–187,791)	245,845 (−4,052–520,883)	3.4409 (3.2542–3.6279)
45–49	1,628 (−1,548–4,769)	6,452 (−178–14,337)	3.7130 (3.4455–3.9812)	72,828 (−67,219–212,422)	290,459 (−6,995–644,653)	3.7366 (3.4705–4.0034)
50–69	14,611 (−7,393–39,216)	38,482 (−1,383–91,102)	2.5694 (2.4087–2.7304)	461,530 (−232,206–1,232,363)	1,237,160 (−37,821–2,863,910)	2.6869 (2.5533–2.8207)
Over 70	4,657 (−4,198–15,112)	13,677 (−2,901–40,034)	2.6733 (2.3687–2.9788)	79,333 (−70,766–249,908)	234,541 (−39,893–650,549)	2.5666 (2.2294–2.9048)
SDI Quintile						
Low SDI	555 (−3,004–3,511)	8,463 (−1,706–22,377)	8.7970 (8.4840–9.1108)	15,728 (−126,584–126,165)	311,457 (−59,848–773,817)	9.3514 (8.9570–9.7473)
Low-middle SDI	1,202 (−2,232–4,619)	8,275 (−2,112–21,294)	6.1450 (6.0358–6.2544)	41,357 (−90,980–166,880)	302,778 (−60,943–740,237)	6.3553 (6.2349–6.4759)
Middle SDI	2,102 (−2,166–6,614)	10,396 (−1,316–24,617)	4.8608 (4.7198–5.0019)	75,171 (−83,171–247,960)	388,123 (−35,758–891,474)	5.0153 (4.8499–5.1811)
High-middle SDI	1,729 (−9,033–11,073)	15,018 (−2,375–36,595)	5.2840 (4.9498–5.6193)	64,256 (−298,338–363,453)	514,755 (−43,029–1,193,974)	5.2181 (4.9044–5.5328)
High SDI	17,638 (−3,133–44,969)	26,452 (487–58,815)	0.5435 (0.2871–0.8005)	545,933 (−103,176–1,355,066)	811,783 (10,620–1,729,374)	0.5204 (0.2650–0.7765)
World Bank income level						
High-income	11,653 (−163–27,283)	13,679 (300–29,894)	−0.1591 (−0.4653–0.1480)	345,815 (−5,702–798,407)	395,032 (5,873–796,416)	−0.2535 (−0.5569–0.0509)
Upper-middle income	8,276 (−15,387–31,372)	30,761 (−4,265–73,914)	3.3585 (3.1792–3.5380)	285,570 (−509,284–1,011,647)	1,055,464 (−64,348–2,415,566)	3.4049 (3.2361–3.5740)
Lower-middle income	2,511 (−4,181–9,617)	19,810 (−4,265–52,252)	6.6720 (6.4500–6.8945)	85,374 (−178,857–344,817)	721,637 (−149,828–1,789,378)	6.8240 (6.5793–7.0693)
Low income	783 (−1,082–2,820)	4,353 (−548–11,065)	5.4700 (5.1085–5.8328)	25,649 (−46,251–99,998)	156,728 (−18,905–387,163)	5.9010 (5.5665–6.2365)
Countries and territories^b^						
China	−13 (−19,205–15,616)	11,521 (−5,900–33,118)	4.8113 (4.3854–5.2389)	9,034 (−610,172–487,053)	378,406 (−156,285–1,018,519)	4.8898 (4.4357–5.3458)
USA	1,699 (27–3,686)	3,672 (63–7,719)	2.4599 (2.2202–2.7001)	53,679 (706–110,672)	108,158 (1,391–212,984)	2.2243 (1.9734–2.4758)
Central Europe, Eastern Europe and Central Asia	9,087 (86–21,675)	8,371 (146–16,786)	−1.1813 (−1.5958 to −0.7650)	275,519 (2,247–645,147)	252,108 (3,301–502,374)	−1.1993 (−1.6182 to −0.7786)
India	−701 (−3,180–569)	4,063 (−4,397–14,013)	9.2945 (8.1581–10.4430)	−24,951 (−114,801–21,290)	150,159 (−133,819–484,761)	9.1595 (8.0820–10.2478)
Indonesia	−79 (−1,622–1,144)	4,103 (−1,262–11,198)	7.0480 (6.7287–7.3681)	−2,678 (−65,927–44,620)	169,149 (−43,794–451,912)	7.6279 (7.2410–8.0162)
Russian Federation	3,402 (36–7,705)	3,316 (61–7,186)	−1.3516 (−2.0059 to −0.6929)	101,383 (901–222,943)	100,827 (1,385–211,470)	−1.2789 (−1.9299 to −0.6235)
Brazil	1,206 (−180–2,975)	2,716 (32–5,763)	2.2627 (2.0859–2.4398)	42,735 (−6,817–106,602)	90,816 (954–187,039)	2.0477 (1.8798–2.2158)
Mexico	440 (5–962)	956 (13–1,892)	2.2531 (2.0661–2.4404)	16,322 (208–35,121)	34,257 (418–64,714)	2.2131 (2.0153–2.4113)

APC, annual percentage change (APC is used to represent the trend of rate. If APC > 0 and lower UI > 0, upward trend; if APC < 0 and upper UI < 0, downward trend).

DALYs, disability-adjusted life years; SDI, socio-demographic index; UI, uncertainty interval.

a Age-standardized DALYs (or death) per 100,000 population.

b The top 8 locations with the highest numbers of DALYs or deaths.

Over the past 33 years, China has been the territory with the highest deaths and DALYs of intracerebral hemorrhage (Supplementary Figure S5). The other regions with the top absolute global burden of high BMI-related intracerebral hemorrhage were Central Europe, Eastern Europe and Central Asia.

## 4 Discussion

High BMI significantly raises the risk of CVDs, with each 1 kg/m^2^ increase in BMI associated with a 10%–15% rise in hypertension risk (17). Individuals with high BMI are more prone to high cholesterol, triglycerides, and high-density lipoprotein cholesterol, which accelerates atherosclerosis and coronary heart disease risk (18). Obesity also alters cardiac structure and function, increasing the incidence of heart failure and arrhythmia (19). The risk of coronary heart disease in obese individuals is directly proportional to the incidence of obesity (20). High BMI not only raises the risk of CVD occurrence but also exacerbates disease severity and treatment difficulty, imposing a heavy burden on personal health, families, and the healthcare system. It increases both direct medical costs, such as hospitalization and medication, and indirect costs, like reduced working hours and labor ability, placing significant pressure on the social economy (21).

Our results show that high BMI has a profound impact on the global burden of CVDs across all age groups, and it has increased significantly from 1990 to 2023, especially in territories and countries with a high SDI and among high and upper-middle income groups in China, India, and Central Europe, Eastern Europe and Central Asia. The incidence of high BMI-related IHD is lower in women than males, likely due to estrogen's protective effect and men's social and psychological burden (22, 23). However, females have higher incidence rates of high BMI-related hypertension, IHD, and intracerebral hemorrhage. This may be due to the decrease in estrogen levels with age, particularly after women reach menopause (24).

Additionally, the death of high BMI-related IHD is highest in the over 70 age group. In certain regions with high SDI, the death and DALYs associated with high BMI-related IHD rank first. In some upper-middle income populations, the death and DALYs related to high BMI-related IHD are rising rapidly. This is closely linked to the natural environment and the social and psychological pressures faced by these populations. However, for high BMI-related HHD, and IS, the death is highest in those over 70 years old, while DALYs are most significant in the 50–69 age group. In this age group, physical function begins to decline, and the metabolic rate slows down, which means the adverse effects of high BMI are more likely to accumulate, greatly increasing their risk (25–29). In high SDI regions and countries, DALYs from BMI-related HHD are higher. However, DALYs show an upward trend among lower-middle income populations. For high BMI-related intracerebral hemorrhage, the death and DALYs are heaviest in the 50–69 age group. Overall, the global burden of high BMI-related CVDs is highest in high SDI.

Regional differences are significant in the burden of CVDs linked to high BMI. Countries and territories like China, India, the USA, and Central Europe, Eastern Europe and Central Asia face a heavy burden, especially in areas with rapid economic growth and Western-style diets, which increase high-calorie food intake and reduce physical activity, leading to higher BMI and CVD incidence (19, 30). In China, urban areas with high obesity prevalence have higher hospitalization and death for CVDs, while regions with traditional healthy lifestyles see a lighter burden of CVD, despite hypertensive patients (31). Additionally, China's aging population exacerbates the burden. In the USA, the high-calorie diet and sedentary lifestyle make obesity a major CVD risk factor (32, 33).

High BMI contributes significantly to the burden of CVDs by triggering metabolic disorders, insulin resistance, inflammation, and oxidative stress, which promote atherosclerosis and endothelial damage (34, 35). It also leads to cardiac structural changes, increasing heart burden and impairing function over time (36). Obesity worsens heart function, elevates the risk of arrhythmia and heart failure, and reduces the heart's energy metabolism, worsening disease progression and prognosis (37, 38). Patients with high BMI face greater treatment challenges, prolonged recovery, and higher death and DALYs due to associated complications.

Preventing and controlling CVDs is challenging, particularly in regions with limited medical resources and low awareness, leading to delayed diagnoses and poor management (39). Global population aging exacerbates the issue, as the older adult are at high risk for CVDs (40). The complex etiology of CVDs, influenced by genetics, lifestyle, and environment, complicates prevention and treatment (41). To reduce the burden of high BMI-related CVDs, it's essential to strengthen health education, raise awareness, and promote lifestyle changes such as balanced diets, exercise, smoking cessation, and alcohol reduction (13, 42–44). In Northeast China, high BMI from unhealthy diets leads to IHD, which can be mitigated by dietary adjustments (45). The Mediterranean diet, rich in nuts, can lower blood pressure, lipids, and glucose, and improve heart health (46).

For individuals with high BMI, regular check-ups are essential to monitor blood pressure, lipids, glucose, and manage CVD risk factors. Obesity can also complicate imaging, hindering the assessment of internal injuries (47). Higher BMI in early adulthood (18–34 years) increases CVD risk, particularly IHD (28). It contributes to early vascular and metabolic issues, such as dyslipidemia and hypertension, accelerating CVD progression (48). Overweight or obese individuals face a higher lifetime risk of CVDs, with BMI strongly linked to heart failure (32). BMI-stratified criteria can improve diagnosis and guide exercise testing for potential heart failure (49). Central fat measurements like waist-to-height ratio are more closely associated with heart failure risks than BMI (50, 51). Fat redistribution to the trunk during adolescence emphasizes the need for both BMI and waist-to-height ratio in early CVD intervention.

Patients with high BMI face increased higher postoperative complications, slower recovery, and poorer neurological outcomes (52, 53). This affects their quality of life and places a care burden on families and society (54). Treatment for HHD includes antihypertensive drugs, metabolic therapies, and weight management, with tailored plans to improve metabolic profiles and reduce IS recurrence. Nutritional support and rehabilitation improve physical function, enhance survival, and reduce societal disease burden. Comprehensive interventions that combine medical and lifestyle factors can significantly reduce disease risk and global burden, improving patient outcomes (55–57).

To prevent and reduce the global burden of CVDs associated with high BMI, targeted interventions tailored to economic status, age, and gender are essential. Low income countries and regions should strengthen free primary-level screening and affordable drug supply, while coordinating efforts to control both infectious and chronic diseases (58). High income countries and regions should build sports-friendly cities, reduce health disparities through healthcare incentives and programs for vulnerable populations (59), and help young people develop healthy habits through campus and workplace interventions (60). Men should control their tobacco and alcohol consumption and adopt a healthy diet (61), while women should avoid extreme dieting and focus on postpartum weight management. For older adults, prioritizing smoking cessation, balanced nutrition, and regular exercise can slow age-related declines in cardiovascular function (62). Additionally, at the global level, equitable access to new drugs such as glucagon-like peptide-1 receptor agonists should be promoted (63), and costs should be reduced through technological innovation to benefit low income countries and regions.

China, as a populous nation with a large population base, has the world's highest number of people who are overweight or obese. The China Nutrition and Chronic Disease Report (2020) indicates that among residents aged 18 and above, the rates of overweight and obesity are 34.3% and 16.4%, respectively, with the combined overweight and obesity rate among adults exceeding half (50.7%). The World Obesity Atlas 2024, released by the World Obesity Federation, indicates that China's overweight and obese adult population is projected to grow at an annual rate of 2.8%. By 2030, obesity-related healthcare expenditures in China are expected to account for approximately 22% of the nation's total medical costs, significantly impacting both livelihood and economy (64, 65). By 2030, the rates of overweight and obesity among adults in China is projected to reach 70.5%. Based on the 2024 edition of the Weight Management Guidelines, China has established a standardized weight management protocol for adults, primarily focusing on diet and exercise. Dietary recommendations for overweight and obese individuals are set at 85% and 80% of intake standards, respectively, based on individual basal metabolic rates and actual energy requirements corresponding to physical activity levels. Staple foods should primarily consist of whole grains, with reduced consumption of high-sugar, high-fat, and high-salt foods. Alcohol intake should be strictly limited, and meals should be consumed at regular times and in consistent quantities. Regarding physical activity, overweight and obese individuals should prioritize fat loss as the primary goal and learn mass maintenance as the secondary goal. This involves engaging in prolonged, moderate-to-low intensity aerobic exercise combined with resistance training, performed once or twice daily (66). The standardized weight management protocols for adults developed in China offer valuable reference points for other developing countries in terms of exercise and dietary recommendations.

This study has certain limitations. Regarding data sources, it primarily relies on a single source—GBD 2023—which has limited sample coverage and insufficient representativeness. Additionally, the data suffers from temporal lag and information gaps, affecting the robustness of conclusions. In terms of analytical methods, the use of before-and-after comparisons and Joinpoint analysis for APC calculations lacks explanatory power regarding deep causal relationships between variables and dynamic evolutionary processes. Regarding research content, specific weight management recommendations were not provided for overweight and obese populations across different genders, age groups, or countries and territories. Given the variations in national contexts, cultural practices, and dietary habits worldwide, we only offered general advice on exercise and diet suitable for most individuals.

In future related research, a hybrid data system combining “primary research + multi-source database integration + big data scraping” can be established to expand sample coverage. Research boundaries should be broadened to incorporate diverse subjects and external influencing factors, while adding cross-temporal and spatial comparison dimensions to deepen core issues. Integrating qualitative and quantitative methods alongside interdisciplinary technologies will enhance the scientific rigor and explanatory power of conclusions.

## 5 Conclusion

High BMI significantly contributes to the global burden of CVDs, with disparities in impact across gender, age, and regions, particularly severe in upper-middle income countries and regions with middle SDI. To combat this, comprehensive measures are needed, including health education, lifestyle promotion, disease surveillance, and early diagnosis and treatment, especially in lower-middle income countries and regions. International cooperation is crucial to achieving Sustainable Development Goal 3.4, reducing premature death from non-communicable diseases. Future research should explore factors influencing the global burden of CVDs due to high BMI, assess prevention measures, and inform global health strategies.

## Data Availability

The original contributions presented in the study are included in the article/Supplementary Material, further inquiries can be directed to the corresponding author.
