# Supplementary material for: The global burden of cardiovascular diseases attributable to high body mass index, 1990–2023
Source: Front Cardiovasc Med. 2026 Feb 13;13:1689708. doi: 10.3389/fcvm.2026.1689708 (PMC12946943; doi:10.3389/fcvm.2026.1689708)
Supplement: Supplementary file 1 [file Datasheet1.docx]

| **Supplementary Table 1** | **The burden attributable to BMI risks in 1990 and 2023 and the temporal trends from 1990 to 2023.** | | | | | |
| --- | --- | --- | --- | --- | --- | --- |
| **Demographics** | **1990 deaths(95 % UI)** | **2023 deaths(95 % UI)** | **Annual percentage change of death rates(95 % CI)** | **1990 DALYs(95 % UI)** | **2023 DALYs(95 % UI)** | **Annual percentage change of DALYs^a^ rates(95 % CI)** |
| **Global** | 1,416,790(729,467-2,131,657) | 3,677,531(2,001,548-5,218,806) | 2.8455（2.7823-2.9087) | 50,097,583(24,182,854-77,040,147) | 135,836,586(66,149,656-199,709,558) | 3.0321(2.9954-3.0688) |
| **Sex** |  |  |  |  |  |  |
| Male | 644,545(350,973-960,496) | 1,736,555(977,276-2,432,671) | 2.9740（2.9130-3.0351) | 23,910,190(12,025,579-36,696,323) | 66,572,491(34,764,976-96,545,707) | 3.1191（3.0889-3.1493) |
| Female | 772,246(373,906-1,180,333) | 1,940,975(1,017,224-2,856,433) | 2.7315（2.6656-2.7975) | 26,187,392(11,720,665-40,326,597) | 69,264,095(31,778,867-102,990,178) | 2.9525（2.9069-2.9981) |
| Age groups |  |  |  |  |  |  |
| 0-14 | 513(218-993) | 356(167-639) | -1.2686(-1.3514 to -1.1857) | 215,945(98,203-383,628) | 255,399(118,360-439,704) | 0.5392（0.4781-0.6003) |
| 15-19 | 98(46-169) | 204(95-377) | 1.9193（1.8250-2.0138) | 50,109(22,432-94,115) | 110,784(50,634-191,813) | 2.2960（2.1642-2.4280) |
| 20-24 | 3,374(1,820-5,202) | 7,064(3,861-10,279) | 2.1728（2.1131-2.2326) | 559,359(240,918-944,820) | 1,249,521(529,557-1,944,737) | 2.5357（2.4429-2.6285) |
| 25-29 | 7,654(4,482-12,605) | 16,589(10,049-24,237) | 2.2533（2.1620-2.3448) | 1,059,088(507,065-1,679,998) | 2,374,902(1,150,225-3,589,539) | 2.5510（2.4542-2.6478) |
| 30-34 | 14,275(8,167-22,442) | 33,566(19,977-46,827) | 2.4279（2.3454-2.5104) | 1,636,839(792,891-2,562,493) | 4,000,548(2,040,155-5,841,778) | 2.6822（2.5979-2.7666) |
| 35-39 | 23,023(13,772-34,649) | 53,670(33,063-74,322) | 2.3154（2.2172-2.4138) | 2,322,274(1,135,441-3,613,272) | 5,817,935(2,975,841-8,413,583) | 2.6662（2.5908-2.7415) |
| 40-44 | 34,815(20,540-52,413) | 88,059(54,778-119,317) | 2.4086（2.2895-2.5279) | 2,914,516(1,475,587-4,448,362) | 7,946,784(4,228,414-11,400,113) | 2.8337（2.7219-2.9456) |
| 45-49 | 51,574(29,162-76,087) | 137,727(85,144-184,960) | 2.7974（2.6108-2.9844) | 3,564,610(1,738,854-5,470,266) | 10,490,176(5,561,592-15,096,596) | 3.2481（3.0631-3.4334) |
| 50-69 | 594,920(317,246-882,921) | 1,411,548(792,804-1,979,192) | 2.6032（2.4498-2.7568) | 24,733,920(11,673,022-37,579,691) | 65,840,331(32,267,289-95,345,112) | 3.0740（2.9605-3.1876) |
| Over 70 | 686,544(337,591-1,072,385) | 1,928,748(1,006,075-2,888,547) | 3.0959（3.0564-3.1355) | 13,040,922(5,927,717-20,697,795) | 37,750,206(17,629,864-57,773,557) | 3.1276（3.0677-3.1876) |
| **SDI Quintile** |  |  |  |  |  |  |
| Low SDI | 71,512(40,688-115,930) | 343,188(208,345-496,141) | 5.0631(4.9684-5.1579) | 2,916,191(1,494,122-4,511,317) | 14,147,060(7,747,471-20,797,609) | 5.1091(5.0178-5.2004) |
| Low-middle SDI | 82,462(45,199-124,028) | 403,980(229,714-580,909) | 5.1608(5.0658-5.2559) | 3,232,331(1,654,072-5,029,809) | 15,870,272(8,131,574-23,097,601) | 5.2052(5.1140-5.2965) |
| Middle SDI | 113,879(65,653-163,424) | 479,784(283,865-666,927) | 4.4784(4.4375-4.5194) | 4,332,363(2,321,546-6,467,258) | 18,294,184(9,732,697-26,125,337) | 4.5230(4.4757-4.5704) |
| High-middle SDI | 203,262(119,239-296,620) | 754,357(413,635-1,078,081) | 4.1087(4.0211-4.1964) | 8,306,414(4,306,922-12,480,960) | 29,598,618(14,645,854-43,564,225) | 4.0126(3.9629-4.0623) |
| High SDI | 943,068(458,434-1,472,741) | 1,691,345(852,526-2,478,064) | 1.5686(1.4864-1.6509) | 31,224,164(13,802,763-48,720,199) | 57,764,913(25,688,062-87,510,779) | 1.7059(1.6372-1.7747) |
| **World Bank income level** |  |  |  |  |  |  |
| High income | 708,922(329,649-1,120,172) | 1,168,683(600,885-1,714,707) | 1.3260(1.2410-1.4112) | 22,551,321(9,507,177-35,351,620) | 37,914,492(16,765,892-57,158,045) | 1.4129（1.3434-1.4825) |
| Upper-middle income | 468,882(264,037-694,634) | 1,317,272(707,673-1,901,614) | 3.0418(2.9646-3.1191) | 18,242,422(9,261,347-28,128,728) | 51,237,682(25,082,198-76,029,308) | 3.1023（3.0526-3.1521) |
| Lower-middle income | 189,201(105,612-289,954) | 1,021,208(593,127-1,486,088) | 5.4901(5.3939-5.5864) | 7,456,869(3,887,218-11,639,874) | 40,128,341(20,774,297-58,351,329) | 5.5065（5.3971-5.6161) |
| Low income | 47,149(27,200-71,134) | 165,450(102,858-231,604) | 3.7815(3.6445-3.9186) | 1,759,796(940,086-2,697,872) | 6,393,136(3,562,578-9,199,790) | 3.9320（3.8043-4.0598) |
| **Countries and territories^b^** |  |  |  |  |  |  |
| Central Europe, Eastern Europe and Central Asia | 306,368(136,116-500,151) | 444,262(209,490-667,465) | 0.7283(0.5226-0.9343) | 9,607,588(4,035,656-15,344,646) | 13,635,252(6,118,136-20,924,847) | 0.6386(0.4520-0.8255) |
| India | 39,133(19,932-66,034) | 396,545(208,681-604,575) | 8.0240(7.6602-8.3890) | 1,747,990(830,043-2,978,612) | 15,987,416(7,769,653-23,894,547) | 7.5876(7.2427-7.9336) |
| China | 153,638(94,207-235,097) | 387,199(208,895-596,122) | 2.5860(2.3284-2.8444) | 6,743,635(3,620,750-10,464,522) | 16,644,677(7,487,512-26,262,052) | 2.6854(2.5691-2.8018) |
| USA | 159,798(76,143-241,214) | 337,646(191,068-469,186) | 2.1687(2.0656-2.2719) | 5,465,453(2,408,287-8,379,810) | 11,875,067(5,742,087-17,324,031) | 2.3618(2.3127-2.4108) |
| Mexico | 30,842(16,727-44,008) | 122,155(68,937-164,533) | 4.2904(4.1996-4.3813) | 1,340,876(688,808-1,900,117) | 4,842,539(2,689,035-6,635,270) | 4.0069(3.9319-4.0819) |
| Indonesia | 16,006(8,619-26,530) | 97,790(52,256-145,681) | 6.0165(5.8623-6.1710) | 731,406(360,551-1,173,652) | 4,359,226(2,191,140-6,496,403) | 5.9203(5.7624-6.0786) |
| **Causes** |  |  |  |  |  |  |
| Cardiovascular diseases | 845,343(447,166-1,298,931) | 1,821,942(1,053,679-2,621,532) | 2.2149(2.1425-2.2874) | 21,569,237(11,314,344-33,056,064) | 46,086,182(25,201,022-66,154,615) | 2.1615(2.0923-2.2308) |
| Chronic respiratory diseases | 29,137(13,155-47,057) | 61,417(26,059-107,585) | 2.1577(2.0193-2.2963) | 1,836,512(865,436-2,945,800) | 4,136,004(1,927,729-6,644,725) | 2.4115(2.3602-2.4629) |
| Diabetes and kidney diseases | 313,847(173,132-452,775) | 1,203,385(729,927-1,669,233) | 4.1600(4.1103-4.2097) | 14,595,406(7,508,271-21,281,288) | 54,654,356(28,618,507-76,811,391) | 4.1457(4.1169-4.1745) |
| Digestive diseases | 19,115(4,150-32,016) | 50,379(12,395-82,715) | 3.1568(2.9289-3.3851) | 1,362,626(334,852-2,516,281) | 2,969,872(797,171-5,158,675) | 2.5624(2.5060-2.6188) |
| Neoplasms | 145,852(64,534-233,328) | 377,808(169,558-578,485) | 2.7966(2.7562-2.8369) | 3,840,010(1,778,297-6,068,466) | 9,530,391(4,393,260-14,384,857) | 2.6450(2.6012-2.6887) |
| Neurological disorders | 23,956(-8,609-107,500) | 107,233(-40,335-430,010) | 4.6643(4.6093-4.7195) | 521,797(-186,332-1,835,518) | 2,187,169(-902,202-7,438,489) | 4.4362(4.4100-4.4624) |
| Respiratory infections andtuberculosis | 39,541(7,466-102,999) | 55,367(11,963-143,131) | 1.1053(0.9516-1.2592) | 1,441,967(262,770-3,905,126) | 2,111,517(417,205-5,648,331) | 1.2123(1.0556-1.3692) |
| Sense organ disease |  |  |  | 60,779(-16,976-153,777) | 230,374(-38,192-553,323) | 4.3008(4.2385-4.3631) |
| Musculoskeletal disorders |  |  |  | 4,869,249(571,690-10,041,679) | 13,930,722(1,703,961-27,299,693) | 3.3804(3.3438-3.4171) |

APC, annual percentage change (APC is used to represent the trend of rate. If APC >0 and lower UI > 0, upward trend; if APC <0 and upper UI <0, downward trend).

DALYs, disability-adjusted life years; SDI, socio-demographic index; UI, uncertainty interval.

a Age-standardized DALYs (or death) per 100,000 population.

b The top 6 locations with the highest numbers of DALYs or deaths.


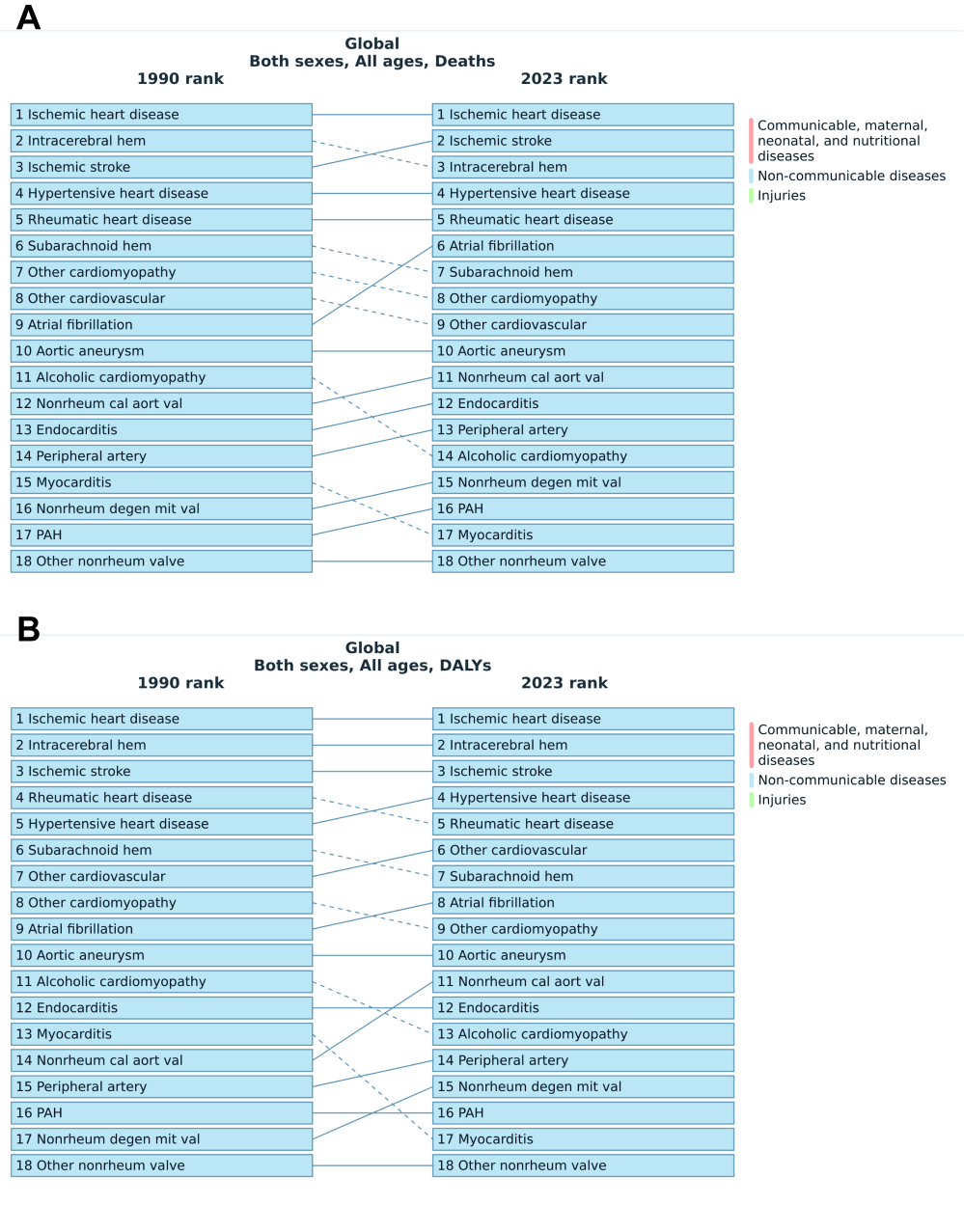


**Supplementary Figure 1. Annual rankings of the number of Deaths and DALYs of CVDs caused by high BMI in 1990 and 2023.** Global rankings of deaths for CVDs in 1990 and 2023(A), and DALYs for CVDs in 1990 and 2023(B).


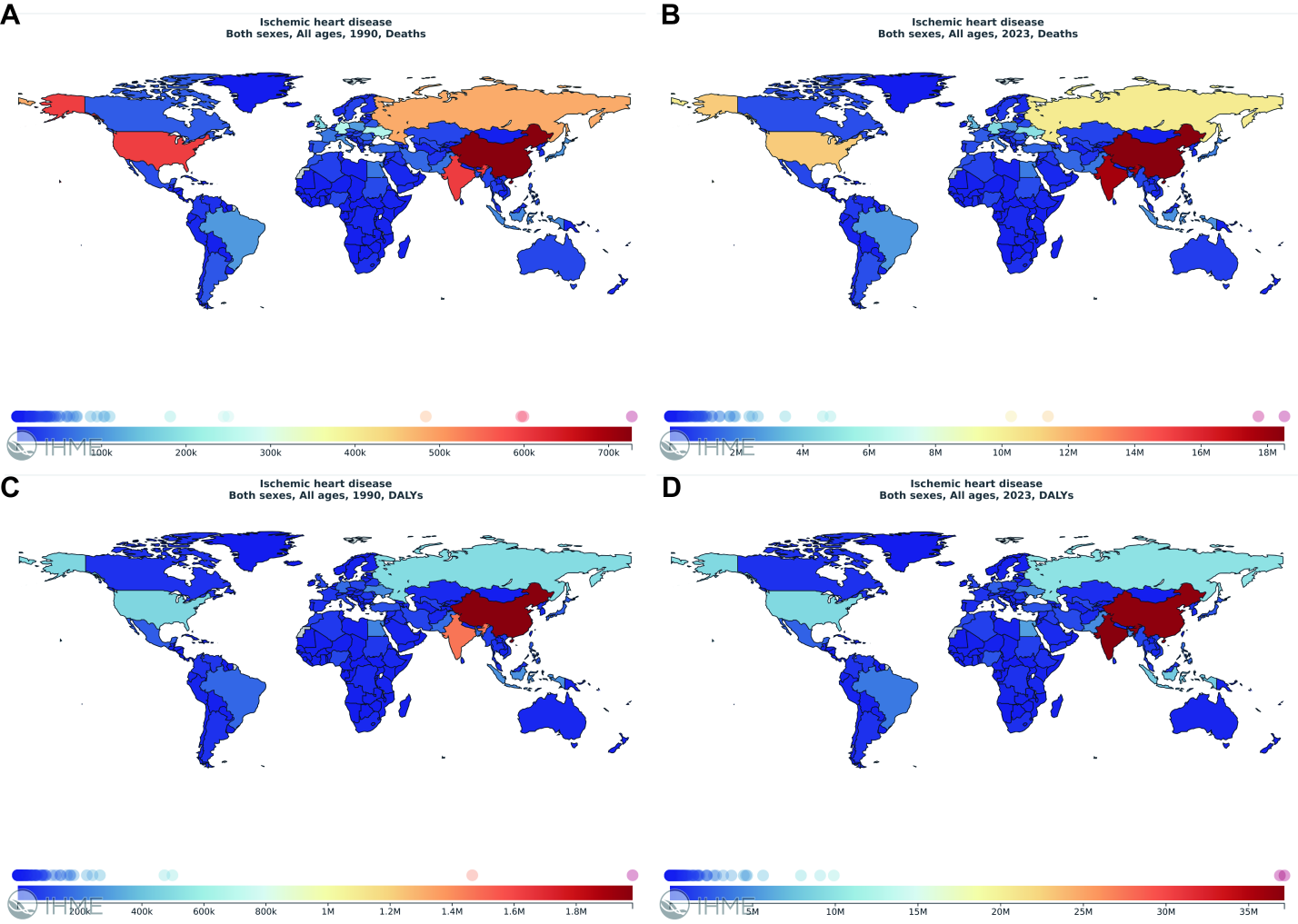


**Supplementary Figure 2. Global distribution of disease burden of high BMI-related ischemic heart disease in 1990 and 2023.** Global maps of ischemic heart disease deaths (A) and DALYs (C) in 1990, and deaths (B) and DALYs (D) in 2023.


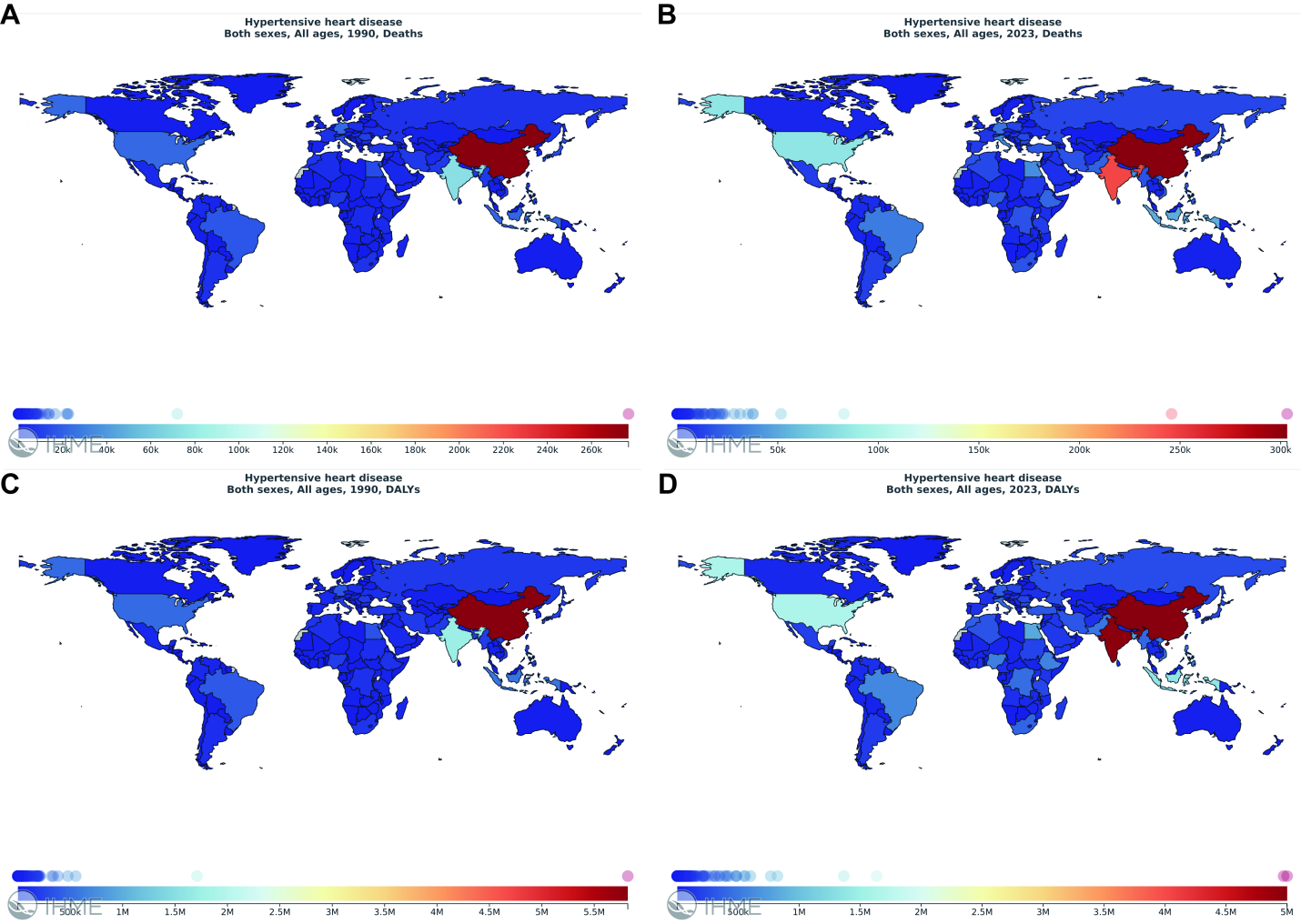


**Supplementary Figure 3. Global distribution of disease burden of high BMI-related hypertensive heart disease in 1990 and 2023.** Global maps of hypertensive heart disease deaths (A) and DALYs (C) in 1990, and deaths (B) and DALYs (D) in 2023.


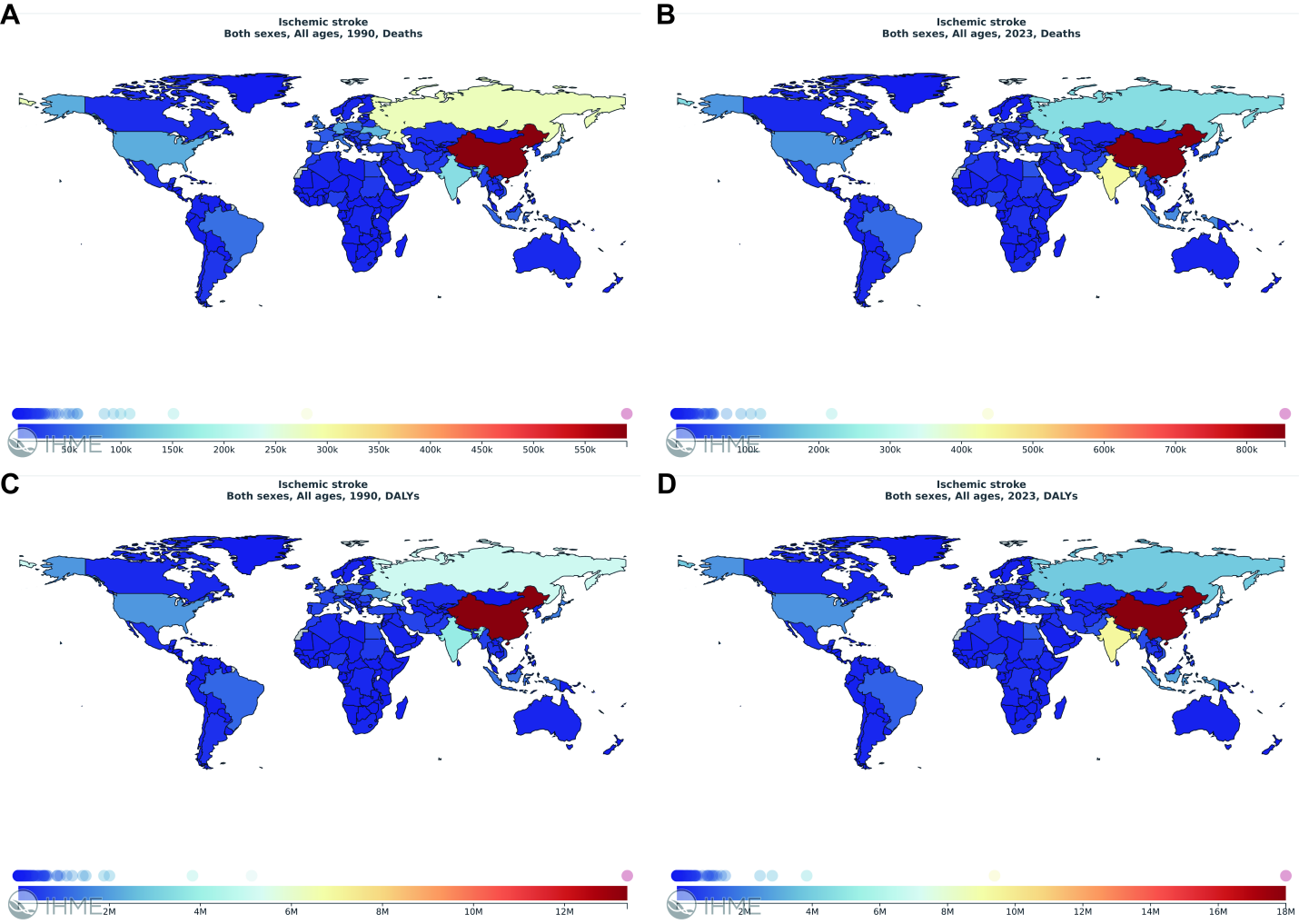


**Supplementary Figure 4. Global distribution of disease burden of high BMI-related ischemic stroke in 1990 and 2023.** Global maps of ischemic stroke deaths (A) and DALYs (C) in 1990, and deaths (B) and DALYs (D) in 2023.


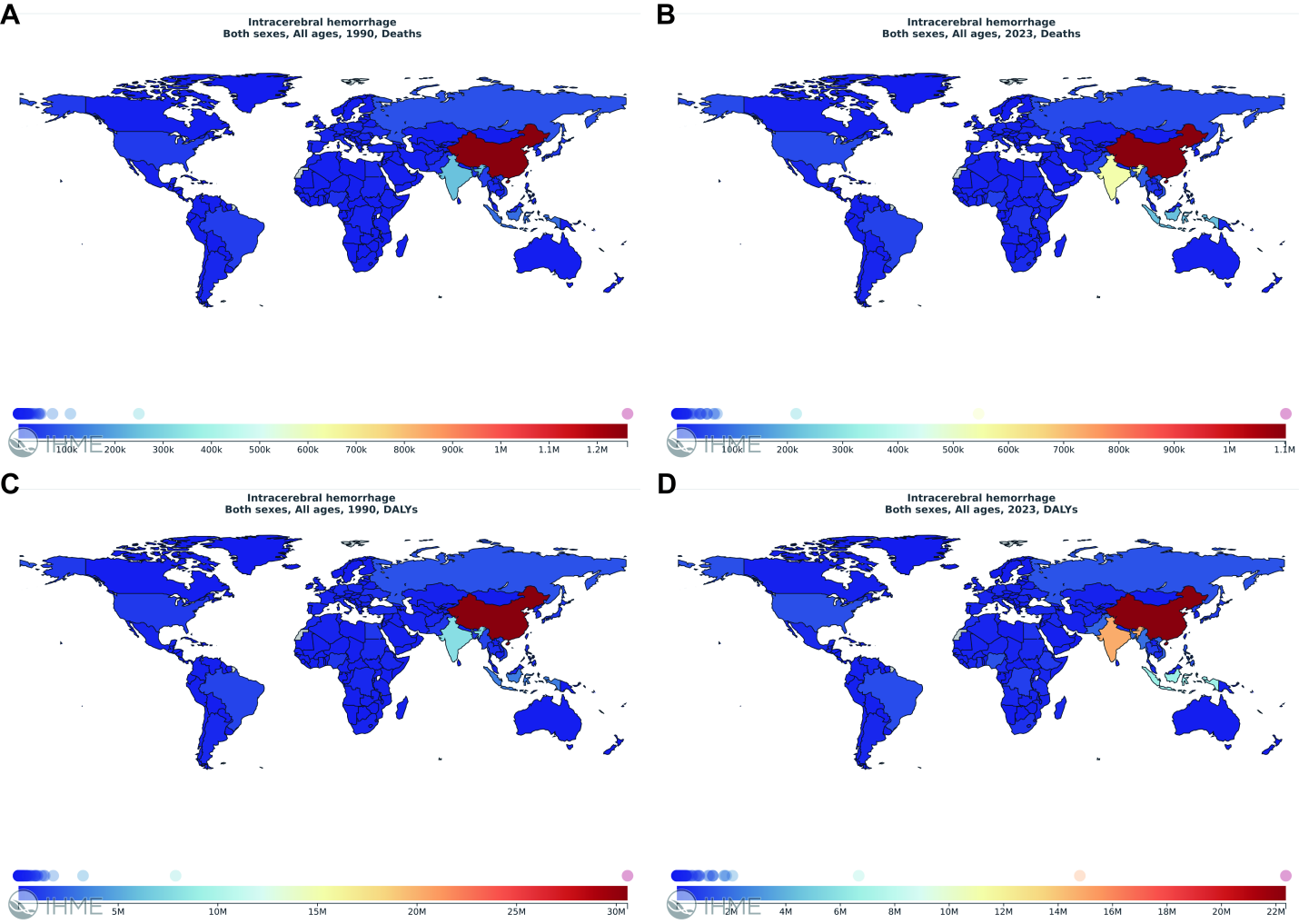


**Supplementary Figure 5. Global distribution of disease burden of high BMI-related intracerebral hemorrhage in 1990 and 2023.** Global maps of intracerebral hemorrhage deaths (A) and DALYs (C) in 1990, and deaths (B) and DALYs (D) in 2023.
